# Growth factor-eluting cochlear implant electrode: impact on residual auditory function, insertional trauma, and fibrosis

**DOI:** 10.1186/s12967-014-0280-4

**Published:** 2014-10-04

**Authors:** Yayoi S Kikkawa, Takayuki Nakagawa, Lin Ying, Yasuhiko Tabata, Hirohito Tsubouchi, Akio Ido, Juichi Ito

**Affiliations:** Department of Otolaryngology-Head and Neck Surgery, Graduate School of Medicine, University of Tokyo, Tokyo, Japan; Department of Otolaryngology-Head and Neck Surgery, Graduate School of Medicine, Kyoto University, Kawaharacho 54, Shogoin, Sakyo-ku, 606-8507 Kyoto, Japan; Department of Biomaterials, Field of Tissue Engineering, Institute for Frontier Medical Science, Kyoto University, Kyoto, Japan; Department of Digestive and Life-style Related Diseases, Graduate School of Medical and Dental Sciences, Kagoshima University, Kagoshima, Japan; Department of Otolaryngology and Head and Neck Surgery, Xijing Hospital, Xi’an, China

**Keywords:** Cochlear implant, Drug delivery system, Gelatin hydrogel, Auditory hair cells, Spiral ganglion neuron, Insulin-like growth factor 1 (IGF1), Hepatocyte growth factor (HGF), Guinea pig, Hearing loss, Insertional trauma

## Abstract

**Background:**

A cochlear implant (CI) is an artificial hearing device that can replace a damaged cochlea. The present study examined the use of growth factor-eluting gelatin hydrogel coatings on the electrodes to minimize inner ear trauma during electrode insertion. Insulin-like growth factor 1 (IGF1) and/or hepatocyte growth factor (HGF) were chosen as the agents to be administered.

**Methods:**

Silicone CI electrode analogs were prepared and coated with gelatin hydrogels. Adsorption/release profile of the hydrogel was measured using ^125^I-radiolabeled IGF. Hydrogel-coated electrodes were absorbed with IGF1, HGF, IGF1 plus HGF, or saline (control) and implanted into the basal turns of guinea pig cochleae (n = 5). Auditory sensitivity was determined pre-operatively, immediately after, and 3, 7, 14, 21, and 28 days post-operatively by using auditory brainstem response (ABR; 4–16 kHz). In addition, histological analysis was performed and auditory hair cell (HC) survival, spiral ganglion neuron (SGN) densities, and fibrous tissue thickness were measured.

**Results:**

Compared to non-coated arrays, hydrogel-coated electrodes adsorbed significantly greater amounts of IGF1 and continuously released it for 48 h. Residual hearing measured by ABR thresholds after surgery were elevated by 50–70 dB in all of the electrode-implanted animals, and was maximal immediately after operation. Thresholds were less elevated after hydrogel treatment, and the hearing protection improved when IGF1 or HGF was applied. Histopathologically, hair cell survival, spiral ganglion cell survival, and fibrous tissue thickness were not different between the experimental groups. No serious adverse events were observed during the 4-week observation period.

**Conclusions:**

Our findings provide the first evidence that hydrogel-coated, growth factor-releasing CI electrodes could attenuate insertional trauma and promote recovery from it, suggesting that this combination might be a new drug delivery strategy not only in cochlear implantation but also in treating clinical conditions characterized by inner ear damage.

## Background

Cochlear implants (CIs) provide auditory perception to individuals with severe to profound sensorineural hearing loss by electrically stimulating spiral ganglion neurons (SGNs) via an electrode array implanted into the cochlea [1]. Recent advances in CI technology have led to the development of a new generation of hearing-preserving CIs with less traumatic electrodes that minimize inner ear trauma during electrode insertion. This is particularly true for those with some residual hearing who may benefit from newly emerging stimulus strategies that employ a combination of electrical and acoustic stimulation. Moreover, insertional trauma can also lead to scar [2] and fibrous tissue formation that could result in impedance increase and residual hearing decrease [3,4].

Two surgical strategies have been used to reduce insertional trauma: soft surgery and targeted drug delivery. However, even with the introduction of minimally traumatic “soft” surgical techniques (reviewed in [5] and [6]) and electrodes that have been modified to reduce intracochlear trauma during their insertion [7], residual hearing is lost or incompletely preserved in one-third of cases [8]. Some researchers have begun to explore the possibility that better hearing preservation may be achieved by the application of protective pharmacological agents to the inner ear at the time of surgery ([9,10] and [11], reviewed in [6]).

Advanced drug delivery systems (DDS) present indubitable benefits for drug administration. Over the past three decades, new approaches have been suggested for the development of novel carriers for drug delivery. Gelatin is a protein obtained by the partial hydrolysis of collagen. This biodegradable, biocompatible, and non-immunogenic compound is commonly used in the biomedical field (e.g., drug delivery vehicles and wound dressings). Gelatin hydrogels can be electrostatically combined with other proteins [12] to form a biodegradable carrier. Incorporated proteins are continuously released by the enzymatic degradation of the gelatin polymers after application.

We have developed insulin-like growth factor 1 (IGF1)- and hepatocyte growth factor (HGF)-containing hydrogels [13] and conducted a series of animal experiments, which revealed that topical growth factor application via gelatin hydrogels significantly improved hearing by protecting auditory hair cells (HCs) against damage caused by intense noise exposure [14,15], drug-induced hearing loss [16], or ischemic injury [17], with no adverse events. IGF1 inhibits apoptosis and promotes cell cycle progression to maintain HC numbers in the injured cochlea [18]. Furthermore, our human clinical trial showed the effectiveness of IGF1 hydrogel treatment in patients with idiopathic sudden sensorineural hearing loss that is refractory to glucocorticoid treatment [19].

In the present study, we examined whether growth factor-eluting hydrogels could be used to reduce the trauma associated with cochlear implantation. CI electrode analogs were coated with IGF1- or HGF-containing gelatin hydrogels and their release profile was measured. Hydrogel electrodes were then implanted in guinea pig ears and cochlear damage was assessed both functionally and histologically.

## Methods

### Biodegradable gelatin hydrogels

The biodegradable hydrogels were prepared as described previously [15,20]. A previous analysis of *in vitro* HGF-release profiles from hydrogels has demonstrated that a hydrogel, isoelectric point (IEP) 5.0, made with 5 wt% glutaraldehyde allows optimal HGF delivery [21]. We therefore used this type of hydrogel in the present study.

### Preparation of electrode samples

Silicone guinea pig CI electrode analogs, 0.38 mm in diameter, were prepared specially for this experiment. Sylgard 184 silicone elastomer (Dow Corning, USA) solution was mixed and degassed and filled in PE20 polyethylene tubing (Clay Adams, USA) and cured at 60°C for 12 h.

Electrode samples were then coated with gelatin hydrogels. Electrode’s silicone surface was treated with corona plasma discharge (100 V, 60 sec) and thereafter gelatin hydrogel (IEP 4.77) was cross-linked with 5 wt% acidic gelatin aqueous solution [13]. In the adsorption of IGF1 experiment, selected electrodes were coated twice to ensure thick coating.

### Adsorption/release of IGF1 from hydrogel-coated electrodes

Preparation of ^125^I-radiolabeled IGF was performed according to the method reported previously [13]. Briefly, recombinant human insulin-like growth factor-1 (rhIGF1, Sigma-Aldrich, USA) was radiolabeled with Iodine 125 (^125^I)-labeled sodium iodide (PerkinElmer, USA) with chloramine T and sodium metabisulfite. Then, a hydrogel-coated electrode (1-cm long) was immersed with 5 μL of ^125^I-labeled rhIGF1 and 1 mL of phosphate buffered saline (PBS) in a test tube for 1 h at 37°C. After 1 h, PBS was completely removed and radioactivity in the supernatant was determined with a scintillation counter (Beckman, USA) to calculate adsorbed IGF1.

To measure *in vitro* release of IGF1, fresh PBS, with or without type I collagenase (Gibco-Invitrogen, USA), was added to the rhIGF1-conjugated hydrogel electrodes. The tubes were gently agitated at 37°C and the buffer was changed periodically at 0.5, 1, 2, 4, 8, 24, and 48 h. The radioactivity of the buffer was measured with a scintillation counter.

### Experimental animals

Four to 9-week-old adult male Hartley guinea pigs (weight, 300–450 g; Japan SLC, Hamamatsu, Japan) served as experimental animals. The Animal Research Committee of the Graduate School of Medicine at Kyoto University approved all experimental protocols (MedKyo14169). Animal care was conducted under the supervision of the Institute of Laboratory Animals at the Graduate School of Medicine, Kyoto University. All experimental procedures were performed in accordance with the National Institutes of Health guidelines for the care and use of laboratory animals. All efforts were made to limit the number of animals used and their suffering.

### Surgical procedure and drug application

Hydrogel-coated electrodes (3.3-mm long) were immersed for 12 h in 10 μL physiologic saline solution with rhIGF1 or recombinant human HGF (rhHGF, Sigma-Aldrich, USA) or mixture of rhIGF1 and rhHGF at a concentration of 0.05 mg/mL or 0.5 mg/mL, respectively. As controls, non-coated electrodes or hydrogel only electrodes (immersed in physiologic saline) were used.

All surgeries were performed by the same surgeon (Y.S.K.) under general anesthesia, that is, intra-muscular injection of midazolam (2 mg/kg) and xylazine (2 mg/kg). After opening the tympanic bulla of the temporal bone, a cochleostomy was performed on the basal turn of the cochlea and the dummy electrode was carefully inserted from the hole. After insertion, the stoma was tightly sealed with bone wax, soft tissue, and fibrin glue.

### Functional analysis

Auditory function was assessed by measuring hearing thresholds of each ear, i.e., auditory-evoked brainstem responses (ABRs) in response to pure-tone stimuli. ABR measurements were performed at frequencies of 4, 8, and 16 kHz before and after surgery, and on days 3, 7, 14, 21, and 28 after electrode insertion. Bioelectrical potentials were recorded using subdermal stainless steel needle electrodes inserted at the vertex (ground), ventrolateral to the measured ear (active) and contralateral to the measured ear (reference). Thresholds were determined from a set of responses at varying intensities with 5-dB SPL intervals and electrical signals were averaged for 1024 repetitions. Thresholds at each frequency were verified at least twice.

### Histological analysis

On day 28 after electrode insertion, animals were deeply anesthetized with midazolam and xylazine, euthanized with pentobarbitone sodium, and transcardially perfused with phosphate-buffered 0.9% saline solution followed by phosphate-buffered 4% paraformaldehyde solution. Temporal bones were harvested and fixed with 4% paraformaldehyde in 0.01 mol/l PBS, decalcified with 4% EDTA, cryoprotected with 30% sucrose solution, and embedded in OCT compound (Tissue-Tek, USA). Cryosections (10-μm thickness) were made and stained with hematoxylin and eosin (HE) or Mallory’s trichrome kit (Sigma-Aldrich).

#### Hair cell survival

Auditory HC survival rate was measured as described previously [22]. The number of remaining HCs at the basal, second, and third turn was counted at least in 6 sections per animal. HCs were counted as present if the cell body and cuticular plate looked intact. We calculated the inner and outer hair cell (IHC and OHC) survival rates of these three turns in each animal by using the following formulae: IHC survival rate% = 100 × [(the number of present IHCs of examined specimens)/(the number of examined specimens)]; OHC survival rate% = 100 × [(the number of present OHCs of examined specimens)/(the number of examined specimens)/3].

#### Spiral ganglion neuron count

SGN counting in Rosenthal’s canals was performed in accordance with a method that has previously been described [23]. An unbiased investigator inspected the collection of mid-modiolar sections generated for each cochlea and determined SGN numbers in the each turn of the cochleae from 6 randomly selected sections that underwent surgery. The cross-sectional areas of Rosenthal’s canals were measured using Image/J software (http://imagej.nih.gov/ij/). SGN densities were then calculated by dividing the number of SGNs by the area. This value was used to reduce the variance caused by differences in the cutting directions among the cochlear specimens.

#### Fibrous tissue thickness

The collagen deposition for each trichrome-stained specimen was determined in at least 6 slides per cochlea, and fibrous tissue thickness was determined by measuring the maximum perpendicular length of fibrous capsule surrounding the implanted electrode.

### Statistical analysis

Adsorption of rhIGF1 into hydrogel-coated or non-coated electrodes was analyzed with one-way analysis of variance (ANOVA) using the Statcel2 application (OMS Publishing, Saitama, Japan). Values of *p* less than 0.05 were considered statistically significant. For interactions that were found to be significant, multiple paired comparisons were further analyzed using the Tukey–Kramer test.

The overall effects of the hydrogel-growth factor application on *in vivo* ABR thresholds were examined with repeated-measures ANOVA. *P* values < 0.05 were considered statistically significant.

All histological measurements (hair cell survival, spiral ganglion cell survival, and fibrous tissue thickness) were analyzed with one-way ANOVA.

## Results

### Adsorption and release profile of IGF1 from hydrogel-coated electrodes

**Figure 1:**
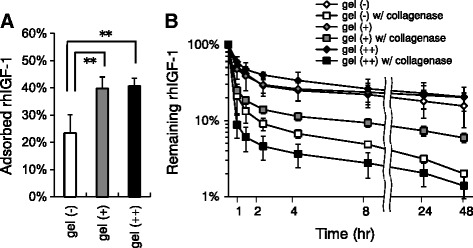
**Adsorption and release profiles of gelatin hydrogel-coated CI electrodes for recombinant human IGF1 (rhIGF1). (A)** Adsorption of rhIGF1 was expressed by relative radioactivities of incorporated ^125^I-labeled rhIGF1 (n = 6). Gel (−), gel (+) and gel (++) represent non-coated, single-hydrogel-coated and double-coated electrodes, respectively. Error bars indicate the standard deviation (SD). *P < 0.01. **(B)** Time-course of the rhIGF1 radioactivity on test electrodes after gently agitated in 37°C phosphate-buffered saline (PBS), with or without type I collagenase. Note that even after 24 h, hydrogel-coated electrodes retained more than 7% of IGF1 in natural environment (gray boxes).

First, we evaluated the adsorption of IGF1 to the synthesized electrodes by radioactivity assay, i.e., measuring relative intensities of radioactivity on the electrodes after they were immersed in ^125^I-labeled IGF1 solution for 1 h. Figure 1A shows adsorption of non-coated bare electrodes and hydrogel-coated samples. Single-hydrogel-coated (gel (+)) electrodes adsorbed 37.6 ± 2.3% (mean ± SD) of given IGF1 in the solution, whereas double-coated (gel (++)) and non-coated (gel (−)) arrays absorbed 39.8 ± 4.2% and 23.4 ± 6.7%, respectively. Both single- and double-coated electrodes showed significant adsorption of IGF1 over bare electrode (P < 0.01). There was no statistical difference between single- and double-coated arrays.

Release profiles of the IGF1 electrodes are shown in Figure 1B. Since human body fluids contain certain amount of collagenase and have gelatinolytic activity, we tested the release of IGF1 from electrodes in collagenase-containing saline. Test samples gave a biphasic release profile with an initial burst release (1 h) followed by secondary slow release phase over the 48-h study period. In collagenase-containing environment, thick-coated electrodes released more drug than other coated or non-coated electrodes (black boxes). Of all three types, single-coated electrodes retained most IGF1 in collagenase fluids, e.g., 7.3 ± 1.1% (mean ± SD) after 24 h (gray boxes).

### Alterations in ABR thresholds after drug-eluting electrode insertion

**Figure 2:**
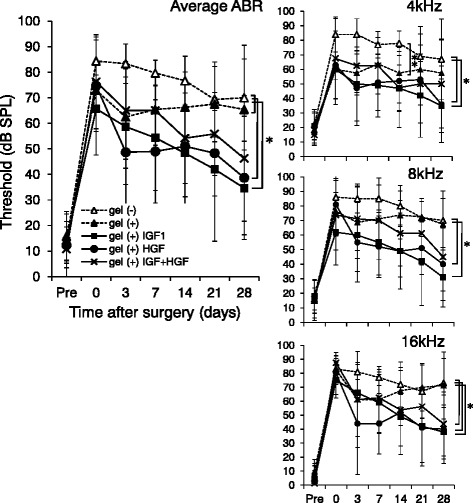
**Alterations in ABR thresholds after drug-eluting electrode insertion.** ABR thresholds at 4, 8 and 16 kHz were obtained before, immediately after surgery and repeated for 4 weeks until the end of the treatment (n = 5 per group). Graphs show ABR thresholds in decibels (mean ± SD) of average of three frequencies (4, 8, and 16 kHz). Repeated measures ANOVA of average ABR thresholds revealed significant differences between non-coated (gel (−)) and IGF1- (gel (+) IGF1) or HGF-adsorbed (gel (+) HGF) hydrogel electrodes (*P < 0.05). Hydrogel-coated electrode without medication (gel (+)) showed superior protective effect over bare electrode. In individual frequency analyses at 4, 8, or 16 kHz, IGF1 or HGF electrode showed significant attenuation of hearing loss after surgery, whereas IGF plus HGF electrode showed significant differences only at 16 kHz. Gel (+) electrode showed significant hearing loss-reducing effect until 2 weeks after surgery in analyses using 4 kHz ABR thresholds (**P < 0.025).

To evaluate surgical damage-reducing effect of these electrodes, changes in auditory function of operated animals were measured. ABR thresholds at 4, 8, and 16 kHz were recorded before, immediately after surgery, and 3, 7, 14, 21, and 28 days post-operatively (Figure 2). Pretreatment baseline ABR thresholds did not differ among the five groups and were consistent with data previously obtained in our laboratory [15]. Our surgical procedure caused average hearing loss around 61 dB, whereas non-coated, hydrogel-coated, IGF1, HGF, and IGF plus HGF electrodes shifted ABR thresholds by 70 ± 14, 57 ± 11, 52 ± 20, 62 ± 21, and 65 ± 26 dB (mean ± SD), respectively. ABR thresholds recovered consistently in IGF1 or HGF electrode groups during the 4-week test period, leading to significant differences over bare electrode at each ABR frequency and their average (P < 0.05). In contrast, IGF plus HGF electrode showed significant difference only at high frequency (16 kHz). Hydrogel-coated electrode without medication showed significant effect after 4 weeks at average ABR; further analyses revealed hydrogel-only group showed reduced hearing loss up until 2 weeks at low frequency (4 kHz, P < 0.025).

### Survival of the HCs

**Figure 3:**
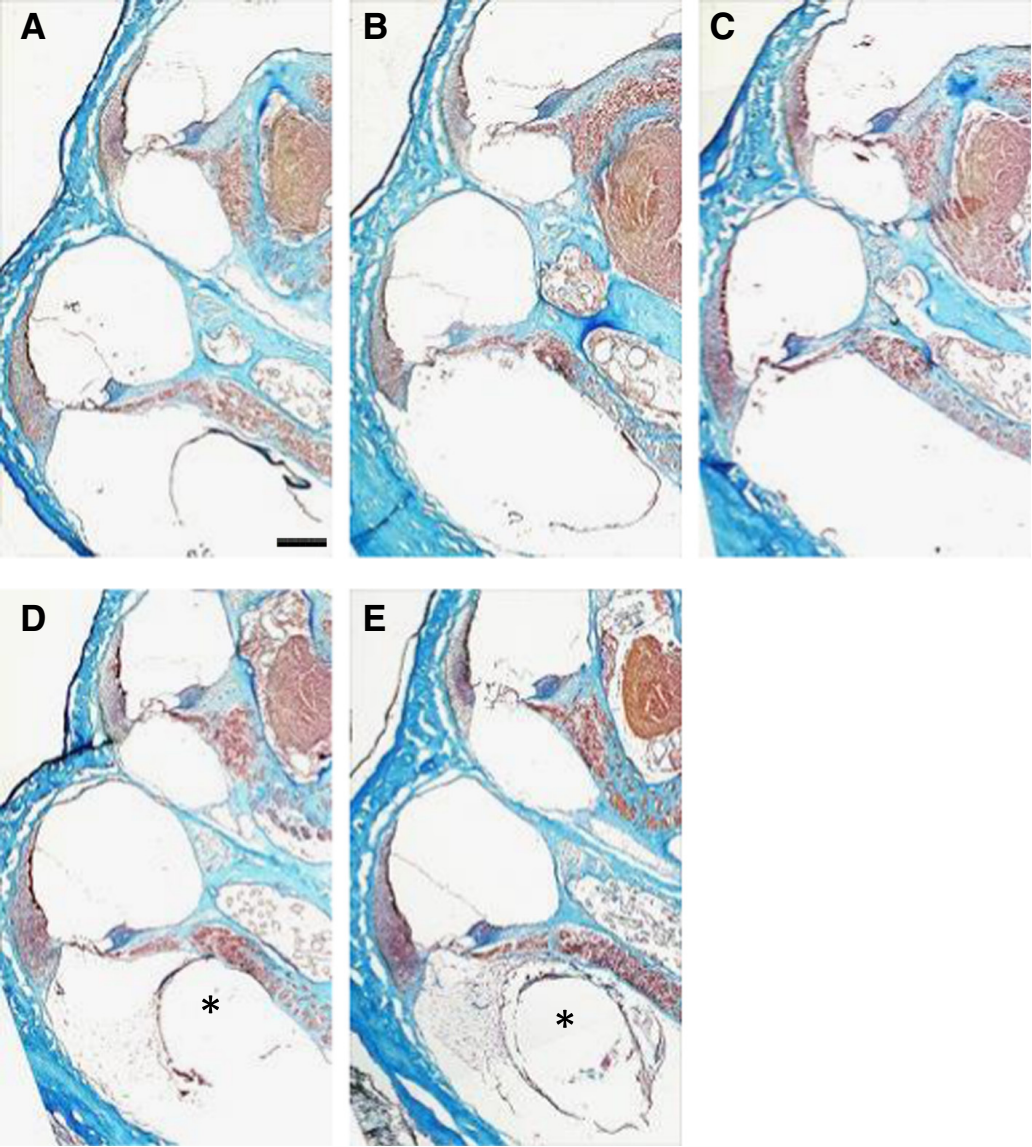
**Representative photomicrographs of the guinea pig specimens.** Mid-modiolar cochlear sections from groups **A** (non-coated), **B** (gelatin hydrogel coating), **C** (hydrogel + IGF1), **D** (hydrogel + HGF) and **E** (hydrogel + IGF1 + HGF). Third and basal turns of the cochlea are shown. In some cases, dense fibrous tissue was observed around the inserted electrode arrays (asterisks). Mallory’s trichrome staining. Scale bar, 250 μm.

**Figure 4:**
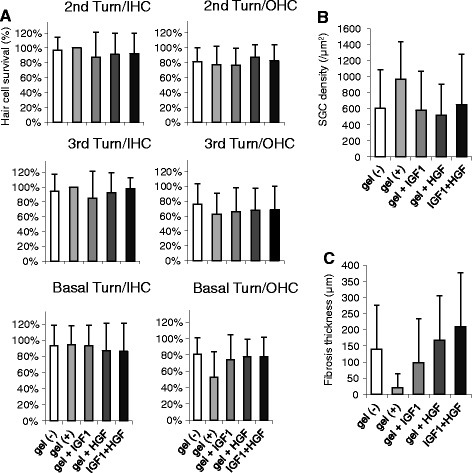
**Survival of the cochlear cells and fibrous tissue formation. (A)** The survival rates (mean ± SD) of the inner (left column) and outer (right column) hair cells in the second, third, and basal turns of operated guinea pigs from each experimental group. **(B)** The survival rates (mean ± SD) of spiral ganglion cells in the Rosenthal’s canal. **(C)** Thickness of the fibrous formation (mean ± SD) of the basal turns from each group.

All guinea pigs displayed loss of outer hair cells (OHC) in all turns, more significantly in the basal turn, whereas IHCs displayed no or only a little loss (Figures 3 and 4A). The total IHC survival rates (mean ± SD) in non-coated, hydrogel-coated, IGF1, HGF, and IGF plus HGF electrodes-inserted guinea pigs were 95.1% ± 7.0%, 98.3% ± 5.8%, 86.1% ± 15.5%, 84.6% ± 26.7%, and 90.0% ± 10.9%, respectively. Although not statistically significant, the IHC survival rates were better at the second and third turns in hydrogel-coated guinea pigs when compared to non-coated counterparts.

In contrast, greater variance was observed in the extent of OHC loss. The total OHC survival rates in non-coated, hydrogel-coated, IGF1, HGF, and IGF plus HGF electrodes-inserted guinea pigs were 77.5% ± 11.6%, 65.6% ± 16.1%, 71.2% ± 17.9%, 76.3% ± 13.9%, and 78.1% ± 12.8%, respectively. In hydrogel-coated electrode GPs, the OHC survival rates in the basal turns were relatively lower when compared to those of non-coated or growth factor-treated ones (not statistically significant). In contrast, there was no significant difference in all GPs at either second or third turns of the cochlea.

### Spiral ganglion cell (SGC) density

We quantified the histological damage to SGNs by measuring SGC densities in Rosenthal’s canals (Figure 4B). The total SGC densities in non-coated, hydrogel-coated, IGF1, HGF, and IGF plus HGF electrodes-inserted guinea pigs were 603 ± 480/μm^2^ and 968 ± 466/μm^2^, 581 ± 483/μm^2^, 520 ± 386/μm^2^, and 649 ± 628/μm^2^ (mean ± SD), respectively. No significant differences in the SGC densities were found, but SGNs were most preserved in hydrogel-coated electrode inserted guinea pigs. In either non-coated, IGF1 or HGF guinea pigs, the SGC densities were relatively lower when compared to those in hydrogel-coated electrode group.

### Fibrous tissue response of implanted cochleae

Fibrous capsule stained with trichrome was observed around the implants and the capsular thickness was measured (Figures 3 and 4C). Out of 22 implanted cochleae, 13 had minor or no fibrous tissue present in histological sections.

The remaining 9 cochleae had relatively dense fibrous tissue occupying the scala tympani in cross section (thickness varying from 160.2 to 338.6 μm). These 9 GPs were found in dummy (non-coated) electrode group or IGF1, HGF, and IGF1 plus HGF groups, but not in the hydrogel-only group. Although not statistically significant, a general trend was found that hydrogel coating tended to suppress fibrous tissue formation compared to bare silicone, which showed biocompatibility of the coating. Growth factor-administered groups showed denser fibrous tissue, but the difference was not statistically significant.

## Discussion

Our findings demonstrate that efficient transfer of neuroprotective growth factors into the inner ear is achievable with a hydrogel-coated CI electrode. Adsorption and release profile analyses in the present study confirmed the sustained delivery of IGF1 to the cochlear fluid for at least 48 h by way of the biodegradable hydrogel. In addition, *in vivo* ABR hearing record indicated that the local application of growth factors to the inner ear is an effective method for the protection of the cochlea from insertional trauma. The functional and morphologic protection of the HC and SGN was observed at 28 days after growth factor application, indicating that its biological effects were successfully maintained during this period. Moreover, no adverse events including functional and histological reaction were observed in treated animals.

Biodegradable hydrogel coating on CI electrodes is relatively straightforward procedure to apply neuroprotective agents to the cochlea compared to systemic or middle ear injection. The inner ear is separated from the systemic circulation by a blood-labyrinth barrier that limits penetration of certain molecules from the systemic circulation to the inner ear. Previous studies investigating the efficacy of neurotrophins against inner ear degeneration have used the implantation of an osmotic minipump [24] or gene transfer by virus vectors [25,26] as drug-application methods. The osmotic minipump requires extra surgery, which includes implantation of the pump into the subcutaneous tissue. Although recent gene transfer studies using virus vectors have shown no significant toxicity, this risk remains a major problem in clinical applications. In contrast with virus vectors, biodegradable hydrogels are made from porcine collagen and have no toxicity [20,21]. We, therefore, consider the biodegradable electrode coating to be better suited for clinical use than the osmotic minipump or gene transfer. Another study demonstrated that polypyrrole/para-toluene sulfonate-containing neurotrophin-3 (Ppy/pTS/NT3)-coated electrode arrays had lower electrically evoked ABR thresholds and greater SGN densities in implanted cochleae [1].

Efforts to reduce degeneration in the inner ear have identified several agents for the protection of HCs or SGNs. Among these are trophic growth factors, several of which are commercially available for clinical use. Our focus was on IGF1, which has been approved for clinical application. In a preceding paper, we found that IGF1 could activate both the PI3K/Akt and MEK/ERK pathways in the neomycin-injured mouse cochlea [18]. The PI3K/Akt pathway maintained the number of IHCs through the inhibition of apoptosis and the MEK/ERK pathway was activated in the Hensen’s and Claudius’ cells and induced the cell cycle promotion that partly contributed to the maintenance of the OHCs. We reported the efficacy of topical IGF1 application for sudden sensorineural hearing loss (SSHL) that is resistant to systemic glucocorticoid treatments in a single arm, non-randomized and open trial [19].

Another promising agent is HGF, which was originally identified as a protein which stimulates proliferation of the hepatocytes. We found that local HGF treatment significantly reduced ABR threshold shifts and loss of outer hair cells in the basal portion of damaged cochleae due to noise exposure [15]. HGF activates ectodermal cell outgrowth in a similar pathway as IGF1 [27]; however, since HGF has different molecular weight (69 kDa for α-subunit and 34 kDa for β-subunit) compared to that of IGF1 (7.6 kDa), HGF may abundantly distribute in the basal portion of the cochlea in comparison with the distribution of IGF1. Therefore, we speculated that a combined approach (IGF1 and HGF) may be beneficial to protect wider portion of the cochlea.

In this study, hydrogel coating alone protected the cochlea from insertional trauma, especially in the lower tone (Figure 2). Hydrogel coating also increased SGC and IHC survival in upper turns of the cochlea, but the differences were not statistically significant. Roland et al. suggested that insertional trauma arises partially from a perilymph fluid leak from cochleostomy [28]. Hydrogels are commonly known as superabsorbents because they can absorb 400 to 1500 times their dry weight in water. Therefore, an interesting possibility arises that hydrogel coating worked as a natural sealant around the insertion site, thus preventing perilymph leakage. However, the protective effect of hydrogel coating was reduced after 4 weeks following surgery.

In contrast, the effect of the growth factors was increased over time. This was most evident in higher tones (16 kHz). OHC survival was most promoted in the basal turns, but the differences were not statistically significant. According to the release profile data (Figure 1B), growth factor release from the electrode rapidly declined even if combined with gelatin hydrogels. Sustained pro-proliferative effect of growth factors in our study might have resulted from the natural sealant effect of hydrogel coating. Nevertheless, fibrous tissue formation was accelerated in the presence of growth factors (IGF1, HGF or IGF plus HGF). Because of the limitation in sample numbers, these results should be fully confirmed with further experiments in the near future.

## Conclusions

Growth factor-eluting gelatin hydrogel coatings for CI electrodes have been described. This coating successfully released growth factors in collagenase-added physiological saline and when implanted into guinea pig cochleae, coated electrodes promoted a significant recovery of hearing compared to plain silicone electrodes, as indicated from lower ABR thresholds. Given the absence of adverse effect in the operated GPs, these electrodes appear promising for clinical use. With atraumatic insertion techniques, they would seem to be well suited for patients who have substantial residual hearing and who would benefit from combined electrical and acoustic stimulation.

## Abbreviations

IGF1: Insulin-like growth factor 1
HGF: Hepatocyte growth factor
CI: Cochlar implant
SGN: Spiral ganglion neuron
PI3K: Phosphoinositide-3-kinase
Akt: Protein kinase B
MEK: Mitogen-activated protein kinase/extracellular signal-regulated kinase kinase
ERK: Extracellular signal-regulated kinase
ABR: Auditory-evoked brainstem response
HC: Auditory hair cells
IHC: Inner hair cells
OHC: Outer hair cells
SSHL: Sudden sensorineural hearing loss
